# Screening, Characterization and Mutagenesis Breeding of *Monascus* Isolates with High Esterification Activity

**DOI:** 10.3390/foods14223949

**Published:** 2025-11-18

**Authors:** Chen Zhou, Shuran Yang, Xingche Zhu, Xiaoxi Li, Jing Li, Zhenghui Lu

**Affiliations:** 1 State Key Laboratory of Plant Diversity and Specialty Crops, Wuhan Botanical Garden, Chinese Academy of Sciences, Wuhan 430074, China; 2 State Key Laboratory of Biocatalysis and Enzyme Engineering, School of Life Sciences, Hubei University, Wuhan 430062, China; 3 Hubei Jiangxia Laboratory, Wuhan 430299, China

**Keywords:** *Monascus*, red koji, esterification activity, mutagenesis breeding

## Abstract

Esters are predominant fragrance components in various traditional fermented foods. Hongqu rice wine, a beverage gaining popularity among young consumers in China, largely owes its aromatic profile to esterases derived from *Monascus* species. However, research on esterification characteristics of *Monascus* strains remains limited, constraining efforts to improve the quality and flavor of Hongqu rice wine. To better understand their esterification characteristics of commercial *Monascus* strains from different regions of China and further develop a high-quality esterifying *Monascus* strain for the liquor industry, we identified five *Monascus* isolates from red koji samples used in rice wine fermentation. Their esterification activity was evaluated by preparing red koji through solid-state fermentation of wheat bran under conditions simulating industrial production. Among the isolates, *M. purpureus* M21 exhibited the highest reported esterification activity to date, reaching 88.5 ± 8.6 U. Through atmospheric and room-temperature plasma (ARTP) mutagenesis breeding, the esterification activity of *M. purpureus* M21 was further enhanced by 41% to 124.8 U. In summary, this study not only figures out the properties of commercial esterifying *Monascus* from diverse regional sources but also significantly enhances the esterification performance of a potent esterifying *Monascus* strain without invoking GMO controversies. This high-performance esterifying *Monascus* strain presents a promising fermentation starter to enhance the flavor profile of Hongqu rice wine and diverse fermented beverages, thereby meeting evolving consumer preferences.

## 1. Introduction

*Monascus* is a genus of filamentous saprophytic fungi belonging to the phylum *Eumycota*, subphylum *Ascomycotina*, class *Plectomycetes*, order *Eurotiales*, and family *Monascaceae*. Currently, it comprises 12 recognized species, including *M. floridanus*, *M. pallens*, *M. purpureus* and *M. ruber* [1]. Its fermentation product, known as Red koji (also referred to angkak, beni koji, or red yeast rice), is rich in various functional compounds. Studies have found that *Monascus* sp. produces a diverse array of pigments, with over 110 pigments having been identified and characterized. Among these, the six most well-characterized pigments include two red pigments (rubropunctamine and monascorubramine), two yellow pigments (ankaflavin and monascin), and two orange pigments (rubropunctatin and monascorubrin) [2]. These pigments have been traditionally used as natural colorants and preservatives in foods including Chinese cheese, bagoong, wine, tofu, sake, miso, pork, sausage and fish [3,4]. Beyond pigments, *Monascus* are known to produce various functional metabolites [5]. Among them, monacolin K (also known as Lovastatin) is the most extensively studied bioactive component, which is considered as the most efficacious 3-hydroxy-3-methylglutaryl-coenzyme A reductase inhibitor [6]. Hence, red koji containing monacolin K has been used as a blood cholesterol-reducing drug for decades. Notably, Studies revealed that the oral bioavailability of lovastatin was improved in red koji products (LipoCol Forte, Cholestin, or Xuezhikang, containing 22.8 mg lovastatin) compared to pure lovastatin tablets (Mevacor or Lovasta, 20 mg), owing to a higher dissolution rate and reduced crystallinity [7]. In addition, *Monascus* produces various enzymes, such as saccharifying and esterifying enzymes [8,9,10], making red koji a valuable fermentation starter in the production of Chinese spirits [11,12].

As corroborated by recent industry reports and market analyses, the global alcoholic beverage market is undergoing a revolution [13,14,15,16]. According to IWSR, the low- and no-alcohol (Lo-No) beverage category exceeded US$11 billion in 2022 and is expected to grow by one-third by 2026, making it the fastest-growing segment of the alcohol beverage market by far [17]. In China, the low-alcohol beverage market is projected to exceed RMB 74 billion in 2025, with a compound annual growth rate of 25% [18]. This surge is primarily driven by younger consumers seeking milder and healthier alternatives to high-alcohol liquors. A survey by Yibin Wuliangye Co., Ltd. (Yibin, China), a well-known Chinese Baijiu manufacturing enterprise, revealed that over 60% of respondents (aged 20 to 35 across China) prefer low-alcohol beverages due to their less pungent and irritating sensation [18]. Although low-alcohol beverages are emerging as a new niche for the industry to explore, some challenges must be acknowledged. For example, alcohol reduction often compromises volatile compounds and overall flavor richness. A survey in South Korea found that 56% of beer drinkers avoid Lo-No beers due to unsatisfactory taste [19].

In traditional Chinese liquor fermentation, flavor esters are mainly formed through two pathways. One well-known process is the esterase-mediated dehydration reaction between alcohols and organic acids. This reaction is favored under liquor fermentation conditions, where solid-state fermentation with ethanol accumulation creates low-water-activity environments [20]. The other involves alcohol acyltransferases, which catalyze the formation of esters from alcohols and acyl-CoA, a process that is important in various yeasts [21]. Despite the importance of microbial metabolism in flavor development, current solutions to improve the flavor of low-alcohol beverages primarily rely on the use of flavor-enhancing additives [22], while the potential of modifying fermentation microorganisms to intrinsically boost ester synthesis during low-alcohol beverage production remains limited.

Chinese rice wine, one of the world’s oldest alcoholic beverages, holds a significant place among traditional Chinese liquors [23]. In recent years, Hongqu rice wine, brewed primarily from glutinous rice using red koji as the fermentation starter [24,25], has become a popular beverage among young consumers in China due to its moderate alcohol content, distinctive red color, nutritional value, and unique flavor profile [26]. The aromatic compounds in Hongqu rice wine are mainly synthesized by esterases produced by *Monascus*, which catalyze the formation of esters from alcohols and acids, contributing fruity, floral, sweet, and nutty notes [27,28]. Although red koji is already industrialized as an esterifying starter to enhance the flavor of fermented foods, the limited availability of *Monascus* strains with high esterification activity remains a bottleneck for further improving the flavor quality of rice wine. Moreover, variations in the esterification characteristics of *Monascus* strains used in different regions hinder the standardized production of Hongqu rice wine. Currently, studies on the esterification properties of *Monascus* are still scarce, representing a critical gap that limits the full exploitation of its potential in flavor enhancement.

This study aimed to develop high-performance *Monascus* strains with high esterification activity through a combination of strain screening and mutagenesis breeding. The overall objective was to create a robust esterification starter that not only facilitates the standardized production of Hongqu rice wine but also produces superior, low-alcohol beverages with intrinsically enriched flavor profiles. To achieve this, we (1) isolated and identified five *Monascus* strains from red koji samples widely used in rice wine fermentation across different regions of China, (2) systematically evaluated their phylogenetic relationships, esterification characteristics, and ethanol tolerance, and (3) performed mutagenesis breeding to generate a mutant exhibiting higher esterification activity.

## 2. Materials and Methods

### 2.1. Materials and Reagents

Red koji samples were collected from major Hongqu rice wine-producing areas in China, including Jianou, Gutian and Nanping in Fujian Province, and Yiwu and Jingning in Zhejiang Province. Peptone and yeast extract FM885 were obtained from Angel Yeast (Yichang, China). Potato dextrose agar (PDA, CM0139B) and beef extract (LP0029B) were purchased from Oxoid (London, UK). Potato dextrose broth (PDB, S25657), Glyceryl tributyrate (S30584) and polyvinyl alcohol (S25454) were purchased from Yuanye (Shanghai, China). The wheat bran (feed-grade) was acquired from a local market. Other chemicals and reagents (analytical grade) were supplied by Sinopharm Chemical Reagent (Shanghai, China). Primer synthesis and DNA sequencing were performed by Sangon Biotech (Shanghai, China).

### 2.2. Preliminary Screening of Monascus Strains from Red Koji Samples

A total of 5 g of red koji powder was weighed and added to a 150 mL conical flask containing 45 mL of 1% acetic acid. The flask was sealed with a sterile cover and shaken at 220 rpm for 1 h. The suspension was serially diluted and spread onto PDA medium. *Monascus* strains are characterized by a white initial growth that turns pink or deep purplish-red as they mature. Their mycelia are multinucleate and form extensive, irregular branches, giving rise to the characteristically fluffy appearance. After incubation at 30 °C for 5 days, the strains were isolated based on the above-mentioned characteristic colony morphology and the microscopic structure of the *Monascus* mycelium.

### 2.3. Identification and Phylogenetic Tree Analysis of Monascus Strains

The *Monascus* strains were inoculated in PDB medium and cultured at 30 °C for 4 days. Genomic DNA was extracted using the Fungal DNA Kit (Omega, Norcross, GA, USA). The ITS regions were amplified using primers of ITS1 (5′ TCCGTAGGTGAACCTGCGG 3′) and ITS4 (5′ TCCTCCGCTTATTGATATGC 3′). The PCR products were electrophoresed, and the target ITS fragments were recovered for sequencing. The resulting sequences were analyzed using the BLAST tool (version 2.17.0) based on the GenBank database. Highly similar sequences were selected and aligned using the ClustalW algorithm, and the phylogenetic tree was constructed using the neighbor-joining method in MEGA11 software. The robustness of the phylogenetic tree was evaluated by bootstrap analysis with 1000 replicates.

### 2.4. Solid-State Fermentation and Esterase Activity Determination

A total of 15 g of wheat bran was placed into a 250 mL conical flask containing 18 mL of 1% lactic acid and sterilized at 108 °C for 20 min. Each of the five strains was pre-cultured in PDA medium at 30 °C and 200 rpm for 4 days. The cultures were then inoculated into the wheat bran at a ratio of 5% (*v*/*m*). The samples were incubated statically at 35 °C and mixed once daily. Sterile water was added as needed throughout the 7-day fermentation period. The resulting fermentation products were red koji. Esterification activities of red koji samples were detected according to the light industry standard of the People’s Republic of China (QB/T5188-2017) [29] with three biological replicates. Briefly, 5 g of powdered dried red koji sample was added to 100 mL of a 20% ethanol solution containing 1% hexanoic acid. The mixture was incubated at 32 °C for 100 h to allow esterification. After gently heating, 100 mL of the distillate was collected. A 50 mL of distillate was mixed with 2 drops of phenolphthalein and neutralized with 0.1 M NaOH. Then, 25 mL of 0.1 M NaOH solution was added in excess. The mixture was kept in the dark at room temperature for 24 h, after which it was titrated with 0.1 M H_2_SO_4_. Esterification activity was calculated based on the formula provided in the standard QB/T5188-2017. One unit of esterification activity (U) was defined as the amount of enzyme in 1 g of red koji that catalyzes the formation of 1 mg of ethyl hexanoate from hexanoic acid and ethanol at 32 °C, over 100 h, and is expressed as mg/(g·100 h).

### 2.5. Ethanol Tolerance Test

The five *Monascus* strains were cultured in PDB medium containing different concentrations of ethanol (0%, 3%, 6%, 9%, 12%, 15% and 18%) at 30 °C and 200 rpm for 7 days. Tolerance was assessed based on the morphology and quantity of mycelial balls obtained from three biological replicates.

### 2.6. Emulsified Screening Medium for Esterification Activity

To enable high-throughput screening of *Monascus* mutants for esterification activity, three types of emulsified screening media were prepared. Their compositions were as follows: Medium A contained 1% yeast extract, 10% sodium chloride, 2% agar, and 0.4% tributyrin. Medium B consisted of 0.3% beef extract, 0.5% sodium chloride, 2% agar, and 0.4% tributyrin. Medium C was prepared by mixing the PDA medium with an emulsion (polyvinyl alcohol: glyceryl tributyrate = 19:1) at a ratio of 4:1. After incubating the strains on these media for 5 days, esterification activity was calculated using the hydrolysis circle (HC) ratio, defined as D/C, where D represents the diameter of the transparent hydrolysis zone and C corresponds to the colony diameter. The rationale is that the *Monascus*-derived extracellular esterases hydrolyze the emulsified tributyrin substrates in the solid medium, generating a transparent ring. As esterification is the reverse reaction of hydrolysis, the observed esterase activity serves as a reliable proxy of the strain’s inherent esterification capacity.

### 2.7. Mutation Breeding by Atmospheric and Room Temperature Plasma Mutagenesis (ARTP) Technology

*M. purpureus* M21 was cultured on PDA medium for 5 days. Spores were harvested and suspended in sterile saline, until the solution turned dark red. Then, 10 μL of spore suspension (1.0 × 10^7^ spores/mL) was spread uniformly on the surface of a sterile slide and dried by aseptic air on a clean bench. The slides were placed in the chamber of the ARTP mutagenesis instrument (Wuxi Tmaxtree, Wuxi, China). Viable counts were determined after 30, 60, 90, 120, 150, 180, 210, 240, and 270 s of treatment to calculate the lethal rate. The lethal rate (%) was calculated as ([U-T]/T) × 100, where U and T denote the colony-forming units (CFU/mL) before and after ARTP treatment, respectively. In this calculation, the term (U-T) represents the absolute number of cells that were killed, while dividing this value by T (the number of surviving cells) provides a normalized measure of lethal rate. Following mutagenesis, the spore suspension was spread onto PDA medium. Single colonies were randomly selected, transferred to emulsified screening medium, and the mutant with the highest HC value was selected for further analysis.

## 3. Results

### 3.1. Screening of Monascus Strains

**Figure 1:**
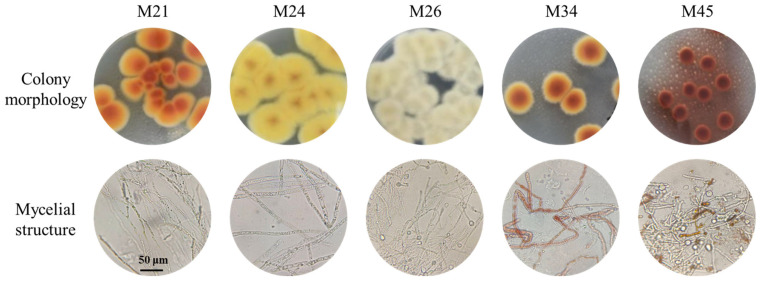
The colony and microscopic morphologies of *Monascus* isolates.

Microorganisms enriched in red koji samples from major Hongqu rice wine-producing regions in China were spread on the PDA medium. *Monascus* stains initially appear white and develop into a pink or purple-red color upon maturation. The mycelia are multinucleate, extensively branched, irregular, and exhibit a fluffy appearance. Based on these traits, five strains, designated M21, M24, M26, M34 and M45, were preliminarily isolated according to colony color and the mycelial structure (Figure 1). After 4 days of cultivation, the colonies of M21 and M34 showed color transitions from yellow at the margins to deep orange in the centers. M24 colonies were yellow with golden-yellow centers. M26 was non-pigmented, and M45 was red with yellow margins. Microscopic examination revealed the presence of cleistothecia and conidia in all five strains.

### 3.2. Species Identification of Monascus Strains

**Figure 2:**
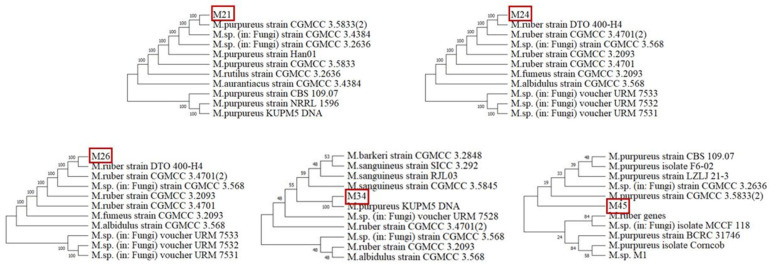
Molecular phylogenetics of *Monascus* isolates based on internal transcribed spacer sequences. The *Monascus* isolates were highlighted with red boxes.

ITS sequences of the *Monascus* strains were compared using the GenBank database via BLAST. All sequences have been deposited in Supplementary Table S1. To determine genetic relationships, highly similar sequences were selected for constructing a phylogenetic tree using the neighbor-joining method of MEGA11 software. As shown in Figure 2, M21 was most closely related to *M. purpureus* strain CGMCC 3.5833. M24 and M26 clustered with *M. ruber*, *M. fumeus*, and *M. albidulus*, showing the closest relationships to *M. purpureus*. M34 and *M. purpureus* KUPM5 clustered into a clade with a strong bootstrap support. M45 was distantly clustered with *M. purpureus*, *M. rutilus*, and *M. aurantiacus*. Combined with the results of morphological and molecular identification, the strains were designated as *M. purpureus* M21, *M. ruber* M24, *M. purpureus* M26, *M. ruber* M34, and *Monascus* sp. M45.

### 3.3. Solid-State Fermentation and Esterase Activity

**Figure 3:**
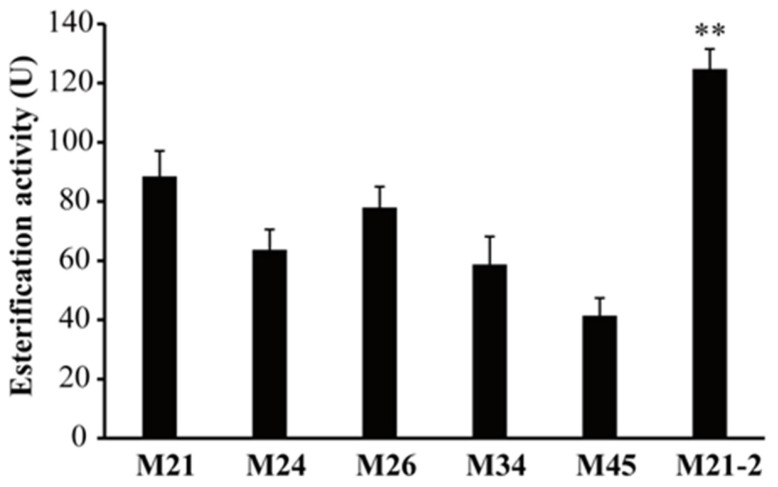
Esterification activity of red koji prepared by solid-state fermentation using *Monascus* isolates and the mutant. The activity was determined according to the method described in the light industry standard of the People’s Republic of China (QB/T5188-2017). All data were presented as mean ± standard deviation (SD, *n* = 3). The significant differences among the wild-type and mutant groups were analyzed by Student’s *t*-test (GraphPad Prism 9). ** *p* < 0.01 vs. M21.

In industrial production, esterifying red koji was produced by solid-state fermentation of *Monascus* using wheat bran as the raw material. The *Monascus* strains M21, M24, M26, M34, and M45 were fermented into red koji, simulating the industrial production process. The esterification activity of red koji was measured according to the light industry standard of the People’s Republic of China QB/T5188-2017. As shown in Figure 3, *M. purpureus* M21 exhibited the highest esterification activity of 88.5 ± 8.6 U, followed by M26 of 78.0 ± 7.1 U. The esterification activity of M24, M34, and M45 was 63.7 ± 6.9 U, 58.8 ± 9.4 U, and 41.5 ± 5.9 U, respectively.

### 3.4. Ethanol Tolerance

The ethanol tolerance was evaluated by culturing the five strains in PDB medium containing different concentrations of ethanol (0%, 3%, 6%, 9%, 12%, 15% and 18%). Based on the morphology and quantity of mycelial spheres, *M. ruber* M34 showed the highest ethanol tolerance, capable of growing in 18% ethanol, although the mycelium became flocculent rather than forming spheres (Table 1). *M. purpureus* M21, *M. purpureus* M26 and *Monascus* sp. M45 tolerated up to 6% ethanol (Table 1). *M. ruber* M24 exhibited the lowest tolerance, with only sparse mycelial spheres observed in 3% ethanol (Table 1).

### 3.5. Mutational Breeding of M. purpureus M21

**Figure 4:**
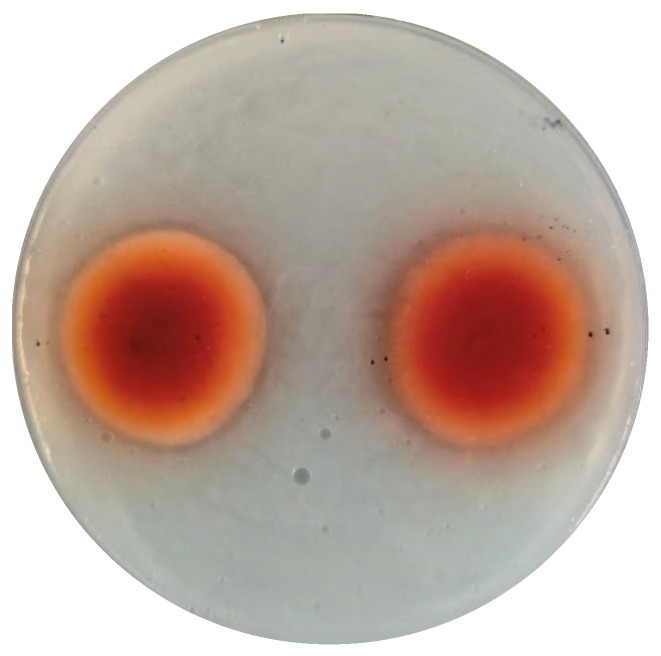
The transparent rings of *M. purpureus* M21 (left) and the mutant M21-2 (right) on emulsified screening medium B after 5 days of cultivation.

Considering its high esterification activity, *M. purpureus* M21 was selected for ARTP mutagenesis. The lethal rate was determined at different treatment durations using the viable count method on PDA medium. Lethal rates of 80–90% were observed after 120–180 s of treatment. Thus, 120 s, 150 s, and 180 s were chosen for mutagenesis. To develop a high-throughput screening method for mutants with enhanced esterification activity, three emulsified screening media (see Section 2: Materials and Methods section) were tested. The results showed that *Monascus* strains could generate a transparent ring only on medium B, where the HC values are positively correlated positively with esterification activities. After ARTP treatment, mutants were inoculated onto the emulsified medium B. One mutant, M21-2, with the highest HC value was selected for the preparation of red koji through solid-state fermentation (Figure 4). The esterification activity of M21-2 reached 124.8 ± 6.7 U, a 41% increase over the wild-type strain (Figure 3). Additionally, the colony of M21-2 was redder than *M. purpureus* M21, indicating that the red pigments production of M21-2 was enhanced (Figure 3). Ethanol tolerance analysis showed almost no difference between M21-2 and *M. purpureus* M21 (Table 1).

## 4. Discussion

Although red koji has been utilized for over a thousand years to enhance flavors of traditional fermented foods such as liquor, rice wine, and vinegar [24,28,30], current research on *Monascus* primarily focuses on pigments and secondary metabolites like monacolin K [31,32,33]. In recent years, esterifying red koji has been industrialized for use in Chinese spirit production, yet studies on the esterification characteristics of *Monascus* remain limited. Chen et al. analyzed the esterase properties of *Monascus* strain Q-306 isolated from Daqu [34]. Zeng et al. reported a correlation between esterase activity and flavor compounds in *Monascus* fermented cheese [35]. It is well established that fermentation conditions significantly influence microbial enzyme production, which is one of the main reasons for the failure of fermentation scale-up. Therefore, the industrial solid-state fermentation conditions were simulated to prepare the red koji of these five newly isolated *Monascus* strains in this study. It was found that the esterification activity of *M. purpureus* M21 reached 88.5 ± 8.6 U, comparable to the highest level previously reported [1].

During wine fermentation, *Monascus* strains face multiple stress factors, among which ethanol toxicity is a major constraint. The ethanol content in Hongqu rice wine typically ranges from 3% to 16%, depending on the fermentation process. Among the five isolates, *M. ruber* M34 grew in the presence of 18% ethanol, while the other strains tolerated only up to 6%. It is worth noting that the ethanol tolerance may be influenced by culture conditions. Guo et al. [36] reported that the *M. ruber* Hm, isolated from fragrant-type liquor Daqu, tolerated up to 9% ethanol in liquid medium, consistent with our findings. However, Zhu et al. [37] observed that nine *Monascus* strains from fragrant-type liquor Daqu could grow in the presence of 21% ethanol on solid agar medium.

The application of genetic modification tools, including transposon mutagenesis, genome-editing technologies, and recombinase systems, in food microorganisms remains limited due to safety concerns and consumer reluctance toward genetically modified organisms (GMOs) [38,39,40]. In contrast, random mutagenesis offers a viable alternative, providing high genetic diversity, independence from prior genomic information, and avoidance of GMO-related controversies, making it particularly suitable for improving traits in food microorganisms. ARTP operates via atmospheric pressure radiofrequency glow discharge, enabling high mutation rates under room temperature conditions conducive to microbial growth [41]. In the present study, ARTP mutagenesis of *M. purpureus* M21 yielded a mutant, M21-2, with a 41% improvement in esterification activity, reaching 124.8 ± 6.7 U.

While random mutagenesis has been widely employed to increase the production of *Monascus* secondary metabolites such as pigments and monacolin K [42,43,44,45]. Its effectiveness largely depends on the availability of efficient high-throughput screening methods, especially for traits that are not visually apparent like enzymatic activity. To address this, we developed an emulsified medium-based assay that enabled rapid and reliable screening of esterification activity. It is worth noting that targeted genome editing tools such as CRISPR/Cas9 have recently been adapted for use in *Monascus*, offering advantages in precision and multiplex genome modification. However, several technical challenges remain, including the low efficiency of homologous recombination in *Monascus*, which makes them prone to randomly integrating foreign DNA at unintended genomic loci, resulting in the off-target phenomenon [46]. Moreover, the limited availability of suitable promoters for sgRNA expression in filamentous fungi further constrains the application of CRISPR systems in *Monascus* [47].

Beyond its practical value, the M21-2 mutant serves as an excellent model for investigating the molecular mechanisms underlying ester synthesis in *Monascus*. Multiple biochemical pathways contribute to flavor ester formation in microorganisms [48,49,50,51], including: (i) esterase-mediated condensation of alcohols and organic acids; (ii) alcohol acyltransferase-catalyzed reaction between alcohols and acyl-CoAs; (iii) alcohol dehydrogenase-mediated reduction of aldehydes or ketones to alcohols, followed by oxidation to esters; (iv) Baeyer–Villiger monooxygenase-driven conversion of ketones to esters or lactones. In *Monascus* strains, esterase is the most crucial enzyme for catalyzing the synthesis of ester compounds. The enhanced esterification activity observed in M21-2 may be attributed to several mechanisms: upregulation of esterase gene expression due to mutations in regulatory regions such as the promoter; improved catalytic efficiency or altered substrate specificity resulting from mutations in the esterase coding region; disruption of native inhibitory regulation; or enhanced enzyme secretion due to modifications in the secretory apparatus.

To fully elucidate the genetic basis of the improved phenotype, omics techniques, such as genome sequencing and transcriptome sequencing, will be essential. These approaches can help identify specific mutations and link them to the observed enhancement of esterifying activity. Furthermore, understanding these genetic alterations opens the door to deciphering the esterification mechanism of *Monascus*. Such knowledge could eventually guide the use of targeted genetic engineering techniques like CRISPR/Cas9 to construct *Monascus* strains with tailored esterification activity without invoking GMO controversies.

## Figures and Tables

**Table 1:** The ethanol tolerance of *Monascus* isolates cultured in liquid media with varying ethanol contents.

	0%	3%	6%	9%	12%	15%	18%
M21	+ + + +	+ + +	+	−	−	−	−
M24	+ + + +	+	−	−	−	−	−
M26	+ + + +	+ + +	+	−	−	−	−
M34	+ + + +	+ + + +	+ + +	+ +	−	−	−
M45	+ + + +	+ + +	+	−	−	−	−
M21-2	+ + + +	+ + +	+	−	−	−	−

Plus sign (+) indicates that the cell can grow, the greater the number of +, the more mycelial spheres there are. Minus sign (−) indicates that the cell cannot grow in that condition.

## Data Availability

The original contributions presented in the study are included in the article/Supplementary Material. Further inquiries can be directed at the corresponding authors.

## Supplementary Materials

The following supporting information can be downloaded at: https://www.mdpi.com/article/10.3390/foods14223949/s1, Table S1: ITS sequences of the *Monascus* strains.
