# Supplementary material for: Screening, Characterization and Mutagenesis Breeding of Monascus Isolates with High Esterification Activity
Source: Foods. 2025 Nov 18;14(22):3949. doi: 10.3390/foods14223949 (PMC12651809; doi:10.3390/foods14223949)
Supplement: Supplementary file 1 [file foods-14-03949-s001.zip › foods-3918342-supplementary.docx]

**Table S1.** ITS sequences of *Monascus* strains

| **Strain** | **ITS Sequence** |
| --- | --- |
| M21 | TTAGAGGAAAAGTAAAGTCGTAACAAGGTTTCCGTAGGTGAACCTGCGGAAGGATCATTACCGAGTGCGGGTCCCCTTCGTGGGACCCAACCTCCCACCCGTGATTATTGTACCTCCTGTTGCTTCGGCGCGGCCCCCTGGGGCCCGCCGGAGACATCTTCTCGAACGCTGTCTTTGAAAAGGATTGCTGTCTGAGTAAACATACCAAATCGGTTAAAACTTTCAACAACGGATCTCTTGGTTCCGGCATCGATGAAGAACGCAGCGAAATGCGATAAGTAATGTGAATTGCAGAATTCAGTGAATCATCGAATCTTTGAACGCACATTGCGCCCCCTGGTATTCCGGGGGGCATGCCTGTCCGAGCGTCATTACTGCCCCTCAAGCGCGGCTTGTGTGTTGGGCCGCCGTCCCCTGCGCCTCCGGGCAACGGGGACGGGCCCGAAAGGCAGTGGCGGCGCCGCGTCCGGTCCTCGAGCGTATGGGGCTTTGTCACCCGCTCAGTAGGTCGGGCCGGGGCCTTTGCCCTCTCCAACCTTTTTTTTCCTTAGGTTGACCTCGGATCAGGTAGGGATACCCGCTGAACTTAAGCATATCAATA |
| M24 | TTCCGTAGGTGAACCTGCGGAAGGATCATTACCGAGTGCGGGTCCCCTTCGTGGGACCCAACCTCCCACCCGTGATTATTGTACCTCCTGTTGCTTCGGCGCGGCCCCCCGGGGCCCGCCGGAGACATCTTCTCGAACGCTGTCTTTGAAAAGGATTGCTGTCTGAGTAAACATACCAAATCGGTTAAAACTTTCAACAACGGATCTCTTGGTTCCGGCATCGATGAAGAACGCAGCGAAATGCGATAAGTAATGTGAATTGCAGAATTCAGTGAATCATCGAATCTTTGAACGCACATTGCGCCCCCTGGTATTCCGGGGGGCATGCCTGTCCGAGCGTCATTACTGCCCCTCAAGCGCGGCTTGTGTGTTGGGCCGCCGTCCCCTGCGCCTCCGGGCAAGGGGGACGGGCCCGAAAGGCAGTGGCGGCGCCGCGTCCGGTCCTCGAGCGTATGGGGCTTTGTCACCCGCTCAGTAGGTCGGGCCGGGGCCTTTGCCCTCTCCAACCTTTTTTTCCTTAGGTTGACCTCGGATCAGGTAGGGATACCCGCTGAACTTAAGCATATCAATAAGCGGAGGAA |
| M26 | TCCGTAGGTGAACCTGCGGAAGGATCATTACCGAGTGCGGGTCCCCTTCGTGGGACCCAACCTCCCACCCGTGATTATTGTACCTCCTGTTGCTTCGGCGCGGCCCCCCGGGGCCCGCCGGAGACATCTTCTCGAACGCTGTCTTTGAAAAGGATTGCTGTCTGAGTAAACATACCAAATCGGTTAAAACTTTCAACAACGGATCTCTTGGTTCCGGCATCGATGAAGAACGCAGCGAAATGCGATAAGTAATGTGAATTGCAGAATTCAGTGAATCATCGAATCTTTGAACGCACATTGCGCCCCCTGGTATTCCGGGGGGCATGCCTGTCCGAGCGTCATTACTGCCCCTCAAGCGCGGCTTGTGTGTTGGGCCGCCGTCCCCTGCGCCTCCGGGCAAGGGGGACGGGCCCGAAAGGCAGTGGCGGCGCCGCGTCCGGTCCTCGAGCGTATGGGGCTTTGTCACCCGCTCAGTAGGTCGGGCCGGGGCCTTTGCCCTCTCCAACCTTTTTTTCCTTAGGTTGACCTCGGATCAGGTAGGGATACCCGCTGAACTTAAGCATATCAATAAGCGGAGG |
| M34 | CTTTTCCCTCCCCTTATTGATATGCTTAAGTTCAGCGGGTATCCCTACCTGATCCGAGGTCAACCTAAGGAAAAAAAGGTTGGAGAGGGCAAAGGCCCCGGCCCGACCTACTGAGCGGGTGACAAAGCCCCATACGCTCGAGGACCGGACGCGGCGCCGCCACTGCCTTTCGGGCCCGTCCCCGTTGCCCGGAGGCGCAGGGGACGGCGGCCCAACACACAAGCCGCGCTTGAGGGGCAGTAATGACGCTCGGACAGGCATGCCCCCCGGAATACCAGGGGGCGCAATGTGCGTTCAAAGATTCGATGATTCACTGAATTCTGCAATTCACATTACTTATCGCATTTCGCTGCGTTCTTCATCGATGCCGGAACCAAGAGATCCGTTGTTGAAAGTTTTAACCGATTTGGTATGTTTACTCAGACAGCAATCCTTTTCAAAGACAGCGTTCGAGAAGATGTCTCCGGCGGGCCCCAGGGGGCCGCGCCGAAGCAACAGGAGGTACAATAATCACGGGTGGGAGGTTGGGTCCCACGAAGGGGACCCGCACTCGGTAATGATCCTTCCGCAGGTTCACCTACGGAAACCTTGTTACGACT |
| M45 | AAGTCGTAACAAGGTTTCCGTAGGTGAACCTGCGGAAGGATCATTACCGAGTGCGGGTCCCCTTCGTGGGACCCAACCTCCCACCCGTGATTATTGTACCTCCTGTTGCTTCGGCGCGGCCCCCTGGGGCCCGCCGGAGACATCTTCTCGAACGCTGTCTTTGAAAAGGATTGCTGTCTGAGTAAACATACCAAATCGGTTAAAACTTTCAACAACGGATCTCTTGGTTCCGGCATCGATGAAGAACGCAGCGAAATGCGATAAGTAATGTGAATTGCAGAATTCAGTGAATCATCGAATCTTTGAACGCACATTGCGCCCCCTGGTATTCCGGGGGGCATGCCTGTCCGAGCGTCATTACTGCCCCTCAAGCGCGGCTTGTGTGTTGGGCCGCCGTCCCCTGCGCCTCCGGGCAACGGGGACGGGCCCGAAAGGCAGTGGCGGCGCCGCGTCCGGTCCTCGAGCGTATGGGGCTTTGTCACCCGCTCAGTAGGTCGGGCCGGGGCCTTTGCCCTCTCCAACCTTTTTTTCCTTAGGTTGACCTCGGATCAGGTAGGGATACCCGCTGAACTTAAGCATATCAAT |
